# The unintended effects of a large minimum wage increase on health: Evidence from South Korea^☆^

**DOI:** 10.1016/j.socscimed.2024.117626

**Published:** 2025-01

**Authors:** Jung Hyun Kim, Marc Suhrcke, Anja K. Leist

**Affiliations:** a University of Luxembourg, Esch-sur-Alzette, Luxembourg; b Luxembourg Institute of Socio-Economic Research, Esch-sur-Alzette, Luxembourg; c Centre for Health Economics, University of York, York, UK

**Keywords:** Minimum wage, Health effects, Older adults, South korea

## Abstract

The 2018 minimum wage increase in South Korea was a major policy change that impacted employment and labour productivity, but its effects on health have not yet been explored. The minimum wage was increased by 16.4% in January 2018, marking the largest increase over two decades and a substantial increase by international standards. While this policy change was a promise of the then-new government, the magnitude of its increase was unexpected. Using a difference-in-differences design with data from the 2016 and 2018 Korean Longitudinal Study on Aging, this study focuses on individuals targeted by the minimum wage policy, particularly older adults earning the minimum wage. Unexpectedly, our results indicate a statistically significant decrease in cognitive function within the targeted group, following the minimum wage hike. However, we did not observe any significant changes in self-reported health. Importantly, for the period 2014 and 2016, when the minimum wage increase was relatively modest, we found positive effects on cognitive health and no negative effects on self-reported health, suggesting that negative effects on cognition emerged only with the large minimum wage increase in 2018. These perhaps unexpected findings may be explained by a significant reduction in the working hours of the targeted group.

## Highlights

• We examined the health effects in older workers from a large minimum wage increase.
• The older workers concerned experienced adverse effects on cognitive function.
• The adverse effects appeared only for the years of the larger minimum wage increase.
• The adverse effects might be attributable to a reduction in working hours.
• We did not observe significant effects on self-reported health.

## 1 Introduction

Minimum wages have long been a highly debated policy measure because of their seemingly complex and ambiguous impacts on wages, employment, and labour productivity. Some argue that minimum wages lead to a decline in working hours and employment for low-waged workers (Neumark et al., 2004), while others claim that it raises the employment probability of low-wage earners as the increased minimum wage is passed on to consumers (Card and Krueger, 1994). While the largest share of existing economic research on minimum wages focuses on labour market effects, for a more comprehensive assessment of the welfare consequences of the policy, it is important to consider other potentially significant social consequences, such as health (Leigh et al., 2019).

In this study, we seek to contribute to the growing but still limited international evidence base on the impact of minimum wages on health. Previous research explored various dimensions of health, including psychological and physical ones (see Leigh et al., 2019 for a review). However, the evidence remains inconsistent, possibly because of varying study designs (Leigh, 2021, Neumark, 2024), data sources, and differences in minimum wage policies. Previous research has also mainly focused on the U.S. and the UK, with insufficient attention given to countries with different economic contexts (Leigh, 2021).

One key aspect that has rarely been studied is the extent to which the size of the increase in the minimum wage matters. For example, a substantial increase in the minimum wage, exceeding 10% in real terms, might have different effects on health outcomes than more modest increases. Maxwell et al. (2022) have most recently compared the health effects of UK minimum wage increases of varying magnitudes across time periods. Their findings indicate that even a sizable minimum wage increase in 2016 (9% in real terms) did not lead to any discernible impact on mental health, aligning with the insignificant effects of the smaller increases. However, it is important to consider that this observed lack of impact might be attributable to the relatively modest increase and that a more substantial wage increase might yield more pronounced effects on health outcomes. The present study aims to fill this research gap by investigating the health impacts of a “large” minimum wage increase on older workers, and specifically for changes in their cognitive functioning and self-reported health. We do so by capitalising on the minimum wage hike in South Korea, which experienced a 16.4% increase (approximately 14.7% of the real growth rate) in 2018. The magnitude of this increase has not been observed in the last two decades in South Korea, and the pace of minimum wage growth has been substantial compared with other countries (Doh and Van der Meer, 2023). This significant minimum wage hike was a promise of the new government that came into power in 2017, although the magnitude of this increase was unanticipated (Doh et al., 2022).

The health implications of the minimum wage are contingent upon its impact on economic factors such as employment, working hours, and earnings. According to Azar et al. (2023), empirical evidence reveals that the labour market effects of the minimum wage vary depending on the concentration of labour. In a monopsonistic setting, where the wage is set below marginal productivity, an increase in the minimum wage does not yield negative employment consequences. In this scenario, the minimum wage signifies an increase in earnings without a decrease in other job-related conditions. Consequently, we may anticipate positive health effects through improved job satisfaction and, thus, better psychological health, at least for those receiving the minimum wage. Nevertheless, some conditions may have adverse health effects, as previous research has demonstrated that increased earnings through the Earned Income Tax Credit (EITC), in the absence of adjusted labour market conditions, results in higher cigarette consumption among lower-income smokers (Kenkel et al., 2014).

Conversely, in a labour market where the wages are already set equal to or higher than the marginal productivity, firms might respond by implementing structural changes to offset potential losses, as associated with the minimum wage increase. In this context, an increase in the minimum wage may lead to adverse employment outcomes, a reduction in working hours, or a substantial increase in workload. Consequently, these structural changes in the workplace may lead to a heightened risk of stress, which in turn may lead to adverse health effects.

Research on the economic impacts of the 2018 South Korean minimum wage hike indicates that the increased minimum wage led to a reduction in total employment (Doh et al., 2022) and gross output (Seok and You, 2022), and an increase in short-term workers who are exempt from social insurance mandates (Kim et al., 2023a). According to Doh et al. (2022), firms responded to a minimum wage increase through layoffs, plant closures, and substitution between labourers of different skill levels. The labour market dynamics in South Korea before the 2018 minimum wage increase resembled a scenario in which wages were already established at levels that did not fall below marginal productivity. In this context, our research aims to investigate how large increases in the minimum wage translate into health outcomes of older workers.

Minimum wage policies are likely to have a greater impact on the health of vulnerable individuals, such as those of advanced ages. In the South Korean context, one in three older adults participates in the labour market after the age of 65, mainly due to a lack of sufficient other income sources (OECD, 2021). In this study, we focus on older adults who continue to work and examine the impact of the minimum wage hike on their physical health and cognitive functioning. We included cognitive functioning as our main health outcome because it is the centre of control of the human body and influences every aspect of life, including an individual’s well-being, development, and productivity. Furthermore, maintaining sound cognitive functioning is essential for effective financial decision-making, which tends to decline after the age of 60 (Finke et al., 2017).

We utilised data from the Korean Longitudinal Study on Aging, which is a sister study of the Health and Retirement Study in the United States. To estimate the impact of the minimum wage increase, we applied a difference-in-differences design. In line with the empirical strategies used in previous studies, our intervention group included individuals whose pre-intervention hourly wages were below the minimum wage, and we compared this group with those whose hourly wages were slightly above the minimum wage (Reeves et al., 2017, Kronenberg et al., 2017, Maxwell et al., 2022).

Unexpectedly, our findings suggest that the minimum wage hike negatively impacted the cognitive functioning of older adults in South Korea, with cognitive function decreasing by 0.704 points during the year of the hike. We did not find statistically significant effects on self-reported health. Between 2014 and 2016, a period during which a modest minimum wage increase was implemented, no negative health effects were found. This suggests that the estimated negative effects are specifically linked to the presence of a larger increase in the minimum wage but not necessarily to smaller increases.

This study builds on previous research exploring the impact of minimum wage on health outcomes, specifically using longitudinal data and a difference-in-differences design to assign intervention groups based on hourly wages (Reeves et al., 2017, Lenhart, 2017, Kronenberg et al., 2017, Maxwell et al., 2022). While some previous studies found positive effects on mental and self-reported health, respectively (Reeves et al., 2017, Lenhart, 2017), others found insignificant results on mental health (Kronenberg et al., 2017) or on both mental and self-reported physical health (Maxwell et al., 2022). Our findings similarly suggest insignificant effects on self-reported health but negative effects on cognitive health for the first time.

This study contributes to the literature in at least three ways. First, we examine the health impact of a minimum wage *hike*. Previous research on minimum wage hikes has focused on economic outcomes, such as unemployment, labour productivity, and gross output (Harasztosi and Lindner, 2019, Doh et al., 2022, Seok and You, 2022, Jardim et al., 2022), but not on health outcomes.

Second, we present evidence from South Korea, which is yet to be investigated in the context of the health effects of the minimum wage. Despite being a high-income country, South Korea’s work environment and corporate culture may differ significantly from those of the U.S., the UK, and Western Europe. Following years of substantial economic growth, the Asian financial crisis of the late 1990s, brought about considerable structural changes and a notable surge in job insecurity (Khang et al., 2005). These significant economic fluctuations within a period of robust growth likely exerted a lasting influence on aspects of both labour supply and demand aspects, such as mandatory early retirement and active labour participation at older ages (OECD, 2018, OECD, 2021). Therefore, evidence from South Korea has the potential to critically enrich the global evidence on the relationship between the minimum wage and health.

Third, we focused on older workers and investigated their cognitive functioning and self-reported health as the main outcomes. Understanding the policy impacts on older workers is particularly important in ageing economies with increasing retirement ages.

The remainder of this paper is organised as follows: Section 2 describes the South Korean context of the minimum wage policy and the labour force participation of older workers. Section 3 describes the data and the methodology used to estimate the intervention effects. Section 4 presents the main results of the study. Section 5 discusses the results and concludes.

**Fig. 1:**
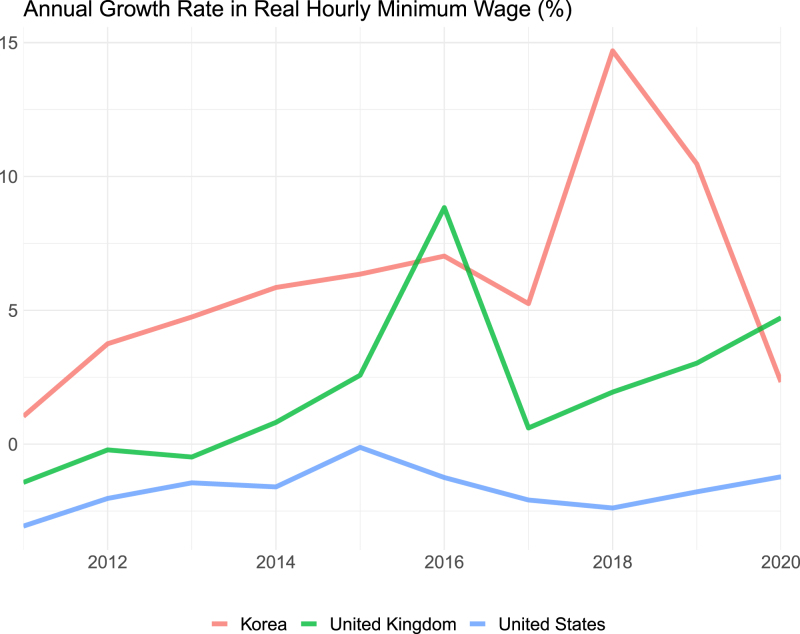
**Annual growth rate in real hourly minimum wages year 2011–2020.** The figure displays international comparisons of the annual growth rate in real hourly minimum wages from 2011 to 2020. As per (OECD, 2023b), real hourly minimum wages are derived by initially deflating the series using the Consumer Price Index, with 2021 serving as the base year. Subsequently, these values are converted into a common currency unit (USD) using Purchasing Power Parities (PPP) for the year 2021. The annual growth levels are then calculated based on these adjusted values.

## 2 Minimum wage and older workers in South Korea

The Minimum Wage Act in South Korea was established in December 1986 and implemented in January 1988 to improve the standard of living of employees, enhance the quality of the labour force, and contribute to the overall development of the national economy (as stated in Article 1 of the Minimum Wage Act). Notably, the minimum wage in South Korea is national and uniform across all industries and business sizes (Jung, 2011), with minor exceptions (as outlined in Article 3 of the Minimum Wage Act). Significant punishment is foreseen for employers who fail to comply with these regulations, such as imprisonment, labour for up to three years, or a fine of up to 20 million Korean won (equivalent to 24,989 USD, adjusted for 2023 Purchasing Power Parities (PPP) according to the World Bank, 2024).

The 2018 minimum wage hike in South Korea was a significant policy change that aimed to improve the standard of living for low-income earners and drive consumption-led economic growth. This increase was the largest since 2001 (Seok and You, 2022), affecting a large part of the population, an estimated 4.6 million individuals, representing 24% of all wage earners in the country (Ministry of Employment and Labor, 2023). Fig. 1 shows the annual growth evolution of the South Korean minimum wage in an international context, comparing it with that of the UK and the U.S. This comparison was made after harmonisation using PPP to account for differences in the cost of living among OECD countries (OECD, 2023b). South Korea stands out because of its significant increase, particularly in 2018, which was the episode we intend to focus on our evaluation. According to Doh and Van der Meer, 2023, the pace of minimum wage growth in South Korea was exceptional compared with other countries, when judged based on the ratio of minimum to mean wages.

South Korea has the highest relative poverty rate among older adults among OECD countries, and the majority of Korean older workers have precarious jobs with non-permanent positions due to mandatory early retirement, which is used to curtail labour costs from the hiring side (OECD, 2021, OECD, 2018). The labour force participation rate of individuals aged 65+ years in South Korea has consistently been greater than 30% from 2012 onwards and was 36% in 2021 (OECD, 2023a), the highest among OECD countries. Many individuals re-enter the labour market after the nominal retirement age because of the low percentage of private occupation-related pensions and insufficient public transfers (Kim et al., 2023b). Among older workers aged 60 and above, 35.1% were estimated to receive wages close to the minimum wage, based on the 2022 national statistics (Korea Minimum Wage Commission, 2023). The within-age group share of minimum wage workers aged 60 and above was the second largest among all age groups, behind only the age group 19 and below.

## 3 Methods

### 3.1 Data

This study utilises data from the Korean Longitudinal Study on Aging (KLoSA), a nationally representative longitudinal survey of over 10,000 individuals aged 45 or older in Korea. Conducted biennially since 2006, the KLoSA has been comparable to the Health and Retirement Study in the U.S. and the English Longitudinal Survey of Aging in the UK (Smith, 2021), providing a valuable dataset for investigating the impact of social and economic policies on older adults. For this study, we focused on waves 2016 and 2018, collected from September to December, as our pre- and post-intervention periods, respectively. One significant advantage of the KLoSA data is the availability of objective measurements of cognitive functioning, enabling us to explore the impact of the minimum wage policy on cognitive health, an crucial factor that has not been investigated. It also provides detailed information on employment types, distinguishing between working for payment, working non-financially, and self-employment. This level of specificity allows us to exclude employment forms that may not be directly affected by minimum wage policies. Moreover, the KLoSA data provides detailed information about the types of pensions individuals receive, which allows for the effective removal of the potential confounding effects of pension benefit status on health.

### 3.2 Intervention and control group assignment

Similar to numerous datasets that offer comprehensive health outcome information, the KLoSA data does not provide precise hourly wage data. Consequently, an approximation is necessary to define the ‘likely’ intervention group, which closely follows the previous research (Reeves et al., 2017, Kronenberg et al., 2017). The selection of the intervention and control groups was based on the derived hourly wage, which was calculated from the self-reported monthly income divided by the number of hours worked (Arulampalam et al., 2004, Reeves et al., 2017, Kronenberg et al., 2017, Bai et al., 2023). A summary of previous studies and the intervention assignments of this study are provided in Table 1.

Individuals with pre-intervention hourly wages below the 2018 minimum wage (7530 Korean Won, equivalent to 8.76 USD when adjusted for 2016 PPP) were assigned to the *intervention group*. In the sensitivity analysis, we put further restrictions on the amount of the wage increase for the intervention group to exclude potentially large wage increases unrelated to the minimum wage policy, which is in line with Reeves et al., 2017.

Individuals with pre-intervention hourly wages between 100%–150% of the 2018 minimum wage are assigned to the *control group*. We conduct sensitivity analyses by varying the upper bound of the pre-intervention hourly wages for the control group in the Table 5. As the derived hourly wages might overestimate the actual hourly wages owing to the lack of information on excess hours, further spillover effects from individuals with slightly higher wages were not considered.

**Fig. 2:**
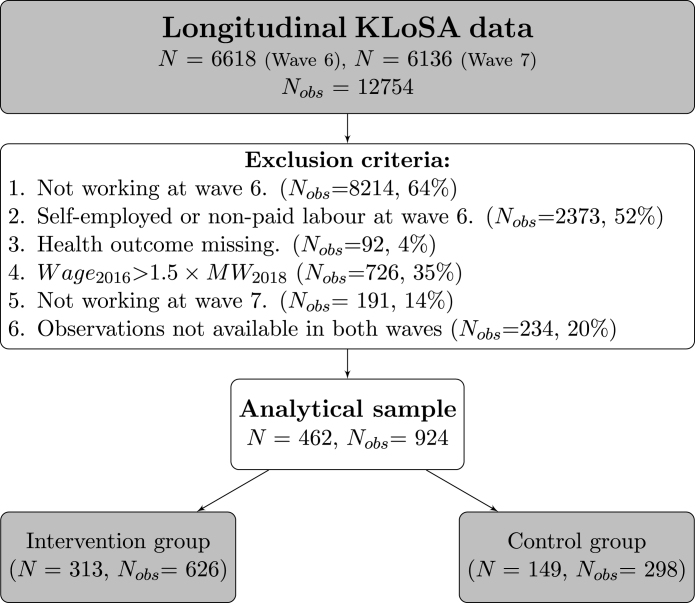
**Flowchart of the final analytical sample** This flowchart summarises the implementation of exclusion criteria to arrive at the final sample. N is the number of individuals; $N_{obs}$ is the number of observations (period-person). The intervention group was defined by participants reporting hourly wages below the minimum wage. Participants reporting hourly wages equal to 100%–150% of minimum wage were chosen as a control group. We also provide estimation results relaxing criterion 5 to include the unemployed individuals in the sensitivity analysis.

Several selection criteria were applied to the samples. First, the sample was limited to individuals who were not self-employed or non-paid labourers during the pre-intervention survey year of 2016. Second, individuals with missing data on the main health outcome were excluded. Third, to create similar characteristics between the intervention and control groups, individuals whose hourly wages were above 150% of the minimum wage were excluded. Fourth, individuals who were not employed during the post-intervention survey year of 2018 were excluded to eliminate the potential unemployment effects. This criterion is in line with the previous research (Reeves et al., 2017, Kronenberg et al., 2017, Maxwell et al., 2022); nevertheless, we also provide the estimation results including unemployed individuals subsequent to the minimum wage hike in the Table 6. Finally, individuals with observations available in both survey years were selected. After applying these selection criteria, a balanced panel of 462 individuals and 924 observations were used to represent the final analytical sample (see Fig. 2).

**Table 1:** Comparisons of intervention assignment.

Studies	Intervention group	Control group	N
Reeves et al. (2017)	(1) $Wage_{pre}$< MW	MW ≤$Wage_{pre}$≤1.1×MW	170
	(2) MW ≤$Wage_{post}$≤1.1×MW		
Kronenberg et al. (2017)	$Wage_{pre}$< MW	MW $<Wage_{pre}$<1.4×MW	1433
Maxwell et al. (2022)	(1) $Wage_{pre}$< MW	MW ≤$Wage_{pre}$≤1.2×MW	803
	(2) MW ≤$Wage_{post}$		
Current study	$Wage_{pre}$< MW	MW ≤$Wage_{pre}$≤1.5×MW	462

*Note.* MW is the national minimum wage for the year. N denotes the number of participants. For the Kronenberg et al. (2017), a wage-based group is used. For the Maxwell et al. (2022), the study sample with the 2016 minimum wage policy is included.

### 3.3 Cognitive functioning and self-rated health outcomes

The primary health outcomes were cognitive functioning and self-reported health status. Cognitive functioning was measured using the Korean version of Mini-Mental State Examination (K-MMSE), a clinically validated tool to measure general cognitive functioning among older adults in Korea (Kang et al., 1997). Scores on the K-MMSE range from 0 to 30, with higher scores indicating better cognitive function. For self-reported health, participants were asked to rate their physical health on a scale of 1 to 5, with higher values indicating better physical health. We also investigated the binary version of self-reported health in Appendix Table S1. We refrained from dichotomising the K-MMSE score due to a lack of a scientific consensus in favour of a specific optimal cut-off across characteristics (Han et al., 2008, Kim et al., 2016).

### 3.4 Statistical model

This study investigated the effects of a minimum wage hike on cognitive functioning and self-reported health. We build on the empirical approach of previous studies that used longitudinal data to assign intervention and control groups based on hourly wages (Reeves et al., 2017, Kronenberg et al., 2017, Maxwell et al., 2022). Specifically, we use two-way fixed effects regressions (TWFE), which produce the same estimation results as the canonical difference-in-differences approach, with two units and two periods (Roth et al., 2023). The adaptation of the two-period difference-in-differences method to study the health impacts of a specific minimum wage policy, based on hourly wage to design the intervention, aligns with the approach used by Maxwell et al. (2022). The regression equation was as follows:

$$Y_{i,t}=\alpha_{i}+\lambda_{t}+\phi X_{i,t}+\left(1\left[t=2018\right]\cdot D_{i}\right)\beta +\epsilon_{i,t}$$

where $Y_{i,t}$ represents the health outcomes (cognitive functioning and self-reported health) at time t. $\alpha_{i}$ and $\lambda_{t}$ are individual- and time-fixed effects, respectively, controlling for time trends in the health outcomes common across individuals. $X_{i,t}$ is a time-varying covariate vector at the individual level, including 5-year interval age dummies to control for non-linear age effects on cognitive and physical health. We also include a dummy variable for marital status and whether individuals receive any of the pensions, including national, specific corporate, or basic pensions. 1[t=2018] is a post-intervention indicator, and $D_{i}$ is the intervention or control group assignment based on the calculated hourly wage before the policy change. Our model specification includes both individual and time-fixed effects. Variables such as education and gender are not included, as they are already accounted for in the individual-fixed effects. Other time-varying factors that apply equally to both the control and intervention groups are also excluded, as they are included in the time-fixed effects. $\epsilon_{i,t}$ is the time-varying error term. Our parameter of interest is $\overset{ˆ}{\beta }$, which measures the average health effect of the minimum wage hike for the intervention group.

Our identification strategy relies on two assumptions: no anticipatory effect and parallel trends. The government announced the new minimum wage in August 2017, which came into effect on 1 January 2018. We assumed that any anticipation of a wage increase or subsequent unemployment would not cause significant changes in work, consumption, or health behaviour, eventually affecting cognitive or self-reported health outcomes before the policy change. The second assumption is that there is a parallel trend in the average health outcomes between the intervention and control groups in the periods preceding the minimum wage hike. Thus, we conducted placebo tests using survey data from 2014 to 2016 to check for pre-existing health impacts on the intervention group before the minimum wage hike.

## 4 Results

### 4.1 Descriptive statistics before the policy change

Table 2 summarises the characteristics of the intervention and control groups measured in 2016, prior to the minimum wage hike. We did not observe any significant differences in cognitive functioning between the two groups. Nevertheless, some variables, such as age, education level, self-reported health, working hours, and monthly income, differed. While the difference in monthly income aligns with the intentional design of the intervention allocation, differences in other pre-intervention characteristics might potentially introduce bias. To mitigate this problem, we conducted a propensity score weighting with regard to age, gender, marital status, education level, self-reported health, working hours, and working days to balance the two groups (Imai and Ratkovic, 2013). After applying the propensity score weights, we failed to detect statistically significant differences in any of these characteristics, except for the monthly income.

We present the characteristics of all individuals below the 2018 minimum wage in 2016 and compare the differences in their subsequent employment statuses in Appendix Table S2. Individuals who were out of the labour market following the minimum wage increase were more likely to be women, older, less educated, report lower levels of health, and perform worse on the cognitive test than those who remained in the labour market.

### 4.2 General effects of the minimum wage hike

This section provides causal evidence of the impact of a minimum wage hike on cognitive functioning and physical health. Results obtained after covariate balancing through propensity score weighting provide the least biased estimates. The minimum wage hike had an adverse effect on cognitive functioning at a statistically significant level of p < 0.05 level but no significant effect on self-reported health as shown in Table 3. With a large minimum wage increase from 2016 to 2018, the workers earning less than the 2018 minimum wage before the policy change experienced an average decline of 0.704 point in cognitive functioning. To understand the size of the impact, obtaining a 5-year consecutive social pension led to a 1.301 point increase in cognitive functioning among older Korean adults using an identical survey and health outcomes to ours (Hwang and Lee, 2022).

**Table 2:** Descriptive statistics at pre-intervention assessment.

Variable	Before weighting		p-value	After weighting		p-value
	Intervention group	Control group		Intervention Group	Control group	
	N=313	N=149		N=313	N=149	
**Health**						
Cognitive function (K-MMSE)	27.36 (2.70)	27.56 (2.96)	0.5	27.50 (2.61)	27.03 (3.30)	0.2
Self-Reported Health:			0.002			0.12
Bad	9.6	2.7		8.3	4.1	
Normal	41	33		39	41	
Good	43	56		45	50	
Very Good	6.1	6.0		7.5	3.6	
Best	0.3	2.0		0.5	1.1	
**Demographics**						
Age	63.90 (6.55)	60.96 (5.39)	<0.001	63.11 (6.45)	62.74 (6.69)	0.7
Female	55	48	0.13	54	56	0.6
Married	83	84	0.9	85	81	0.5
Education:			0.002			>0.9
≤ Elementary school	32	20		28	30	
Middle school	26	19		25	23	
High school	35	48		39	39	
≥ College/University	6.7	13		8.4	8.3	
**Work characteristics**						
Working hours (week)	43.14 (17.98)	40.42 (11.30)	0.048	41.95 (17.22)	40.09 (13.63)	0.3
Working days (week)	4.87 (1.11)	5.01 (0.99)	0.2	4.87 (1.09)	4.94 (1.12)	0.6
Monthly Income (10,000KRW)	101.88 (42.80)	157.23 (45.96)	<0.001	101.20 (42.75)	154.95 (54.26)	<0.001

*Notes*: Listed values are the mean (SD) for continuous variables; otherwise, they are %. This table describes the means of the observable characteristics when comparing the intervention and control groups. The intervention group comprised participants who reported hourly wages below the minimum wage. Participants reporting hourly wages equal to 100%–150% of the minimum wage were selected as the control group. Listed values are mean (standard deviation) for continuous variables and percentages otherwise. Propensity score weights were applied to weighted group. P-values indicate whether statistical differences exist between the two groups. All values were measured in 2016, prior to the large minimum wage increase. 10,000KRW in 2016 is equivalent to 11.64 USD when adjusted for 2016 PPP. K-MMSE: Korean version of the Mini-Mental State Examination.

**Table 3:** Minimum wage and health.

Dependent variable:	Before weighting		After weighting	
	Cognitive function	Self-rated health	Cognitive function	Self-rated health
Minimum wage increase	−0.672	−0.001	$-0.704^{*}$	−0.057
	(0.343)	(0.076)	(0.289)	(0.067)

Observations	924	924	924	924

*Notes*: This table presents an estimate of the impact of the 2016 to 2018 minimum wage increase on health outcomes. The intervention group comprised participants who reported hourly wages below the minimum wage. Participants reporting hourly wages equal to 100%–150% of the minimum wage were selected as the control group. Cognitive scores range from 0 to 30, and self-rated health from 1 to 5. Columns 2–3 report the unadjusted estimation results. Columns 4–5 show propensity-weighted estimation results. All specifications used time-varying variables such as pension status, marital status and 5-year age categorisation. The standard errors are reported in parentheses. *p<0.05, **: *p*<0.01, ***: *p*<0.001.

**Table 4:** Minimum wage and health — Placebo group.

Dependent variable:	Before weighting		After weighting	
	Cognitive function	Self-rated health	Cognitive function	Self-rated health
Minimum wage increase	0.282	0.129	0.494${}^{*}$	0.004
	(0.257)	(0.071)	(0.250)	(0.072)

Observations	990	990	990	990

*Notes*: This table presents an estimate of the impact of the minimum wage on health outcomes of a modest increase from 2014 to 2016, which served as a placebo test. The intervention group comprised participants who reported hourly wages below the minimum wage. Participants reporting hourly wages equal to 100%–150% of the minimum wage were selected as the control group. Cognitive scores range from 0 to 30, and self-rated health from 1 to 5. Columns 2–3 report unadjusted estimation results. Columns 4–5 show propensity-weighted estimation results. All specifications used time-varying variables such as pension status, marital status and 5-year age categorisation. The standard errors are reported in parentheses. *p<0.05, **: *p*<0.01, ***: *p*<0.001.

### 4.3 Sensitivity analysis

#### 4.3.1 Parallel trends assumption and event study results

To assess the validity of the parallel trends assumption, we conducted placebo tests using data from 2014 to 2016, when the minimum wage increased modestly, compared with the large minimum wage increase from 2016 to 2018. In contrast with the reform period, the placebo test showed positive effects on cognitive health and null effects on self-reported health. The findings from the placebo tests suggest that the negative health impacts on cognitive functioning exist only with a considerable increase in the minimum wage (see Table 4). We provide the trends in mean cognitive functioning and self-reported health for both the intervention and control groups in Appendix Figures S1 and S2. This provides confidence that the parallel trends hold and that the decline is only observed in cognitive functioning, not in self-reported health.

**Fig. 3:**
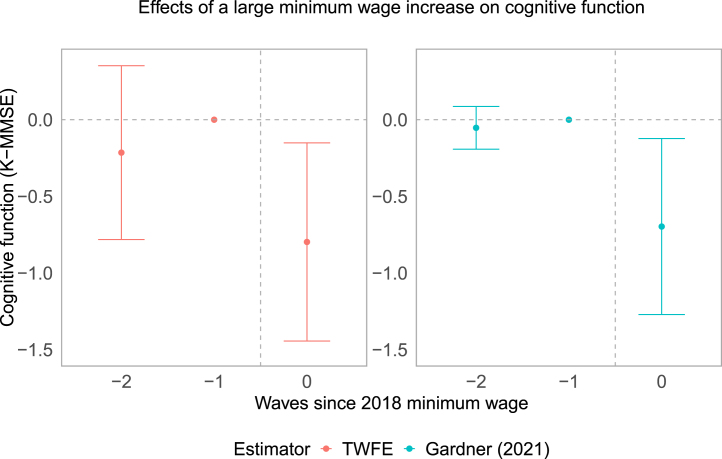
**Event study results regarding cognitive functioning** These figures present the dynamic effects of the large minimum wage increase from 2016 to 2018 on cognitive functioning. The left panel shows the results from using a two-way fixed effects estimator, while the right panel displays the results from the two-stage difference-in-differences estimator. Cognitive function was measured by the Korean version of Mini-Mental State Examination (K-MMSE). K-MMSE ranges from 0 to 30. The intervention group comprised participants whose reported hourly wages in 2016 were below the new minimum wage. The control group included participants whose hourly wages in 2016 were between 100%–150% of the new minimum wage.

Furthermore, we show the event study results of the minimum wage effects on cognitive functioning in Fig. 3 and on self-reported health in Appendix Figure S3. For the event study plot, we included one additional prior wave, from the year 2014. We used the 2016 wage to categorise the treatment and control groups. In this process, 16 individuals who did not participate in 2014 were removed, giving us a balanced panel of 446 individuals and 1338 observations. Due to the potential bias from heterogeneous effects coming from the negative weights (Sun and Abraham, 2021), we used the two-stage difference-in-differences estimator(Gardner et al., 2024) alongside the traditional TWFE. We observe that the results are significant on the year of minimum wage hike and not appearing before. The results from event study strengthen the main results.

#### 4.3.2 Alternative definitions of control groups

As noted in Maxwell et al. (2022), there is a trade-off between the size of the control group and the upper limit of the hourly wage used for the selection. To balance the number of individuals in the two groups in our main analysis, we set the upper limit to 150%. However, reducing the upper limit would have resulted in a more similar control group, albeit with a smaller sample size. To test the sensitivity of our results, we lowered the limit stepwise by 10% and found statistically significant negative effects on cognitive functioning across all upper limits (between 150% and 120%) except the lowest (110%). We provide the results in Table 5. While the smaller sample size of the control group in these scenarios comes with the benefit of greater similarity to the intervention group, our findings suggest that the negative effects on cognitive functioning remain robust across a range of upper limits.

**Table 5:** Alternative definitions of control groups.

Dependent variable	Cognitive functioning				
Control group upper limit:	150%	140%	130%	120%	110%
Minimum wage hike	$-0.704^{*}$	$-0.884^{***}$	$-0.862^{**}$	$-0.625^{*}$	−0.532
	(0.289)	(0.266)	(0.272)	(0.271)	(0.275)

Observations	924	908	866	790	704

*Notes*: This table presents an estimate of the impact of the minimum wage increase from 2016 to 2018 on cognitive functioning. The intervention group comprised participants who reported hourly wages below minimum wage. Participants reporting hourly wages equal to 100%–150% of the minimum wage were selected as the control group. Propensity score weights were then applied. Column 2–6 show a decrease in the control group’s upper limit of 10% of the minimum wage. Column 6 is the smallest control group with individuals with hourly wages from 100%–110% of the minimum wage, and column 2 is the largest one with 100%–150%. All specifications used time-varying variables such as pension status, marital status and 5-year age categorisation. The standard errors are reported in parentheses. *p<0.05, **: *p*<0.01, ***: *p*<0.001.

#### 4.3.3 Alternative definitions of intervention groups

We conducted two sensitivity analyses using different definitions for the intervention groups. The first employs a stringent definition by imposing an additional constraint on the magnitude of the wage increase for the intervention group to rule out a substantial wage increase unrelated to the minimum wage policy. This additional constraint is consistent with Reeves et al., 2017. The intervention group was limited to individuals whose new hourly wage after the policy change was between 100%–120% of the new minimum wage and whose pre-intervention hourly wages were below the new minimum wage (see Appendix Table S4 for the descriptive statistics). Appendix Table S5 demonstrates that these results align with the main findings and, in fact, exhibit even greater strength.

A second sensitivity analysis was conducted, including unemployed individuals, after the minimum wage increase. We relaxed the Exclusion Criterion 5 (Fig. 2) and included individuals who exited the labour force. Skipping the exclusion criterion 5 and subsequently removing observations not available in both waves ($N_{obs}$ = 75, 6%) resulted in an analytical sample of N=637 and $N_{obs}$=1274 where the intervention group had a sample size of N=436 and the control group N=201. Table 6 presents our findings. When we included individuals outside the labour force, we observed negative effects on cognitive functioning solely in the sample with a significant minimum wage increase. Even in the presence of negative impacts of unemployment, the findings are consistent with the main results.

**Table 6:** Minimum wage and health — Including unemployed.

Dependent variable:	Original sample		Placebo test	
	Cognition	Self-rated health	Cognition	Self-rated health
Minimum wage increase	$-0.573^{*}$	−0.019	0.311	0.044
	(0.241)	(0.059)	(0.244)	(0.061)

Observations	1274	1274	1344	1344

*Notes*: This table presents an estimate of the impact of the minimum wage increase on health outcomes. The intervention group was defined as participants reporting hourly wages below the minimum wage, regardless of their subsequent employment status. Participants reporting hourly wages equal to 100%–150% of the minimum wage were selected as the control group. Propensity score weights were then applied. Cognitive scores range from 0 to 30, and self-rated health from 1 to 5. Columns 2-3 report large minimum wage increase from 2016 to 2018. Columns 4–5 show a modest increase from 2014 to 2016, which served as a placebo test. All specifications used time-varying variables such as pension status, marital status and 5-year age categorisation. Standard errors are reported in parentheses. *p<0.05, **: *p*<0.01, ***: *p*<0.001.

### 4.4 Potential mechanisms

A significant increase in the minimum wage may impact health outcomes through two channels: structural changes in the workplace and increases in income. Regarding the first mechanism, it is important to consider not only the increase in the net value of hourly wage but also the associated structural changes (Clemens, 2021). Evidence from Korean data investigating a minimum wage hike founds that there was a substitution between labour at different skill levels (Doh et al., 2022). Similarly, another study from Hungary showed that substantial minimum wage increases led to the substitution of labour with capital (Harasztosi and Lindner, 2019). These findings suggest that a minimum wage hike may trigger structural changes in the labour market, which may have affected job characteristics and work demand. Such a substitution might lead to reduced working hours for low-skilled/inexperienced minimum wage workers and increased cognitive load at work for high-skilled/experienced minimum wage earners. Based on our data, after applying propensity score weighting, we found a statistically significant decrease in working hours by 4.06 h per week for the intervention group compared to the control group after the minimum wage hike (in Appendix Table S3). We investigated other potential mechanisms, including changes in job satisfaction and job security, but found no significant effects. Decreases in working hours may have led to decreases in cognitive or social stimulation at work, but the exact pathways cannot be tested in this dataset, and thus they remain unclear. Future research can empirically test the changes in non-wage-related aspects, although objective measurements of work environment, work-related stress, working conditions, or fringe benefits are often lacking (Clemens, 2021).

Regarding the second proposed mechanism, we observed an increase in monthly income of 174,600 KRW (equivalent to 204 USD when adjusted for 2018 PPP) for the intervention group compared to the control group after the minimum wage hike (in Appendix Table S3). Between 2016 and 2018, when the nominal minimum wage increased by 25%, the intervention group experienced a 34% increase in monthly income, while the control group saw a 12% increase. Some studies argue that an increase in income from a rise in the minimum wage leads to unhealthy behaviours, such as an increase in cigarette smoking and a decrease in fruit and vegetable consumption (Huang et al., 2021, Bai et al., 2023, Andreyeva and Ukert, 2018). Using the South Korean 2018 minimum wage increase as a natural experiment, one study showed that a large minimum wage increase led to an increase in smoking behaviour among working age (19–64 years) study participants (Bai et al., 2023). However, in our analyses adjusted for confounders, we did not observe any statistically significant change in smoking or drinking status within our sample (Appendix Table S3). However, we may have missed more fine-grained changes in the number of cigarettes smoked or units of alcohol consumed due to a lack of information in this dataset. Another study examining the impact of continually receiving social pensions on South Korean older adults, using the same survey and health outcomes as ours concluded that there were positive effects (Hwang and Lee, 2022). Therefore, the pathway through consumption requires further investigation.

We conclude that based on the information and reasoning presented above, the negative effects we found might be driven by changes in non-wage-related job attributes (e.g., change in working hours) from firms actively compensating for the presumed economic losses due to the significant increase in the minimum wage.

## 5 Discussion and conclusion

This study investigates the impact of a substantive minimum wage increase on the cognitive functioning and self-reported health of older workers in South Korea using substantive and given the magnitude of unexpected minimum wage policy changes as a natural experiment. By analysing nationally representative longitudinal individual-level data, we found that the minimum wage hike had a negative impact on cognitive functioning, with a decrease of 0.704 points compared with the unaffected group, while we did not observe significant effects on self-reported health. The magnitude of the cognitive decline is sizable when compared to a study that investigated the health effects of continuous receipt of social pensions among South Korean older adults using the same survey and cognitive measurements, which found a 1.301 points benefit. We also tested for placebo effects by examining the impact of a modest minimum wage increase from 2014 to 2016, and found no negative effects on self-reported health and positive effects on cognitive health. In periods with modest wage increases, individuals earning the minimum wage may experience improved living standards due to additional income, without facing significant negative changes in their working conditions. However, during periods of drastic minimum wage hikes, the advantages of extra income may become less significant when weighed against potential substantial alterations in working conditions. Additional analyses that invalidate possible competing pathways suggest that changes in non-wage-related job attributes, such as decreases in working hours, may have driven the negative impacts. Overall, our study provides important insights into the potentially negative health consequences of a large minimum wage increase.

We investigated the hypotheses proposed by Kronenberg et al., 2017, Maxwell et al., 2022 to determine whether significant minimum wage increases have more pronounced impacts on health outcomes than smaller ones. Our findings suggest that a minimum wage hike may have a negative impact on the cognitive functioning of older workers, whereas modest increases have positive effects. While we attribute these negative impacts to a significant decrease in the working hours of low-wage individuals upon the implementation of the minimum wage hike, the relationship between reduced working hours and cognitive decline requires further investigation. Further research is needed to empirically examine the changes in non-wage aspects due to substantial minimum wage increases, using objective measurements (Clemens, 2021). Although no study has examined the effect of a minimum wage hike on health outcomes, this study adds to the existing literature on the recent minimum wage spike in South Korea and its negative consequences on employment and workers (Doh et al., 2022, Seok and You, 2022, Kim et al., 2023a).

Our study has several limitations that should be considered. First, we need to assume parallel trends between the intervention and control groups. To mitigate potential bias, we used propensity score weighting with pre-intervention characteristics. In addition, a visual inspection of the trends in mean cognitive function for the intervention and control groups (see Appendix Figure S1) and event-study plot in Fig. 3 suggest that the parallel trends assumption is likely to hold. Second, our sample size was small due to the assignment of intervention groups based on hourly wages, which is less biased but has more variance than other criteria, such as education attainment cutoffs. Due to the limited sample size and, consequently, a lack of power, we refrained from further analyses to test heterogeneous effects, e.g., by gender, which may be relevant to consider in future studies. However, these limitations are not unique to our study and apply equally to the previous studies (Reeves et al., 2017, Maxwell et al., 2022). Third, there may have been measurement errors in our calculated hourly wages based on self-reported working hours and income. Our data did not provide information on overtime, which could have resulted in a slight overestimation of hourly wages. Four, there might be wage spillover effects on our control group. Lee and Park (2023) demonstrated the presence of wage spillover effects of minimum wage policy in the Korean labour market up to two times the new minimum wage, affecting 0.3 to 0.5 percentage points of the wage growth rate. Therefore, our estimation results of the minimum wage effects on health might be underestimated. This limitation applies to the previous studies with similar intervention designs (Reeves et al., 2017, Kronenberg et al., 2017, Maxwell et al., 2022). Finally, we cannot rule out that other unobserved time-varying factors (e.g., concurrent economic policies or social changes) may have affected the health of older workers in the intervention and control groups differentially despite their similarities.

Further research could explore the long-term effects of substantial minimum wage increases on health outcomes; however, it is important to consider that current minimum wage policies are not independent of past decisions. When a country experiences a rapid increase in the minimum wage, it may subsequently decrease the minimum wage growth rate in subsequent years, which can confound the long-term health outcomes. Thus, investigating the long-term effects of rapid minimum wage increases can be challenging. Future studies should also confirm the generalisability of our findings by exploring the experiences of other countries that have experienced or are currently facing similar significant minimum wage increases. For instance, Spain, Mexico, and Germany have recently experienced substantial real minimum wage increases. Comparing the impacts of significant minimum wage increases on health outcomes across different countries in varying economic contexts can provide valuable insights and help generalise the findings. The health effects of minimum wage increases are partly dependent on the economic context and need to be considered in cross-national and period-specific changes in minimum wage policies.

Our results cannot be generalised to younger age groups or countries with different economic contexts. Moreover, we cannot assume that the negative health impacts of the minimum wage hike observed in South Korea will continue. Despite these limitations, we advise that caution is necessary when implementing sudden and significant minimum wage increases, as these may have immediate negative effects on older workers’ cognitive functioning through structural changes in the labour market.

## CRediT authorship contribution statement

**Jung Hyun Kim:** Writing – review & editing, Writing – original draft, Methodology, Formal analysis, Conceptualization. **Marc Suhrcke:** Writing – review & editing, Methodology, Conceptualization. **Anja K. Leist:** Writing – review & editing, Supervision, Funding acquisition, Conceptualization.

## Ethics approval

Ethics approval is not required. KLoSA data were approved by Institutional Review Board of Korean Centers for Disease Control and Prevention. KLoSA databases are open for scientific use. Participants gave informed consent to participate in the study before taking part.

## Declaration of competing interest

The authors have no conflicts of interest.

## Data Availability

The code used in this paper is accessible in the Zenodo digital repository at https://doi.org/10.5281/zenodo.14500811.

## Appendix A Supplementary data

MMC S1 .
