# Supplementary material for: The unintended effects of a large minimum wage increase on health: Evidence from South Korea
Source: Soc Sci Med. 2025 Jan;365:117626. doi: 10.1016/j.socscimed.2024.117626 (PMC11803299; doi:10.1016/j.socscimed.2024.117626)
Supplement: MMC S1 — . [file mmc1.pdf]

Table S1: Minimum wage and binary version of self-reported health.

	Self-reported health	
	Before weighting	After weighting
MW increase	0.025	−0.019
	(0.049)	(0.044)
Observations	924	924

Notes: This table presents an estimate of the impact of the 2016 to 2018 minimum wage increase on health outcomes. The intervention group comprised participants who reported hourly wages below the minimum wage. Participants reporting hourly wages equal to 100-150% of the minimum wage were selected as the control group. Self-reported health was re-coded into binary variable with fair/good cutoff. Column 1 report the unadjusted estimation results. Column 2 show propensity-weighted estimation results. All specifications used time-varying variables such as pension status, marital status and 5-year age categorisation. The standard errors are reported in parentheses. *$p<0.05$, **: $p<0.01$, ***: $p<0.001$.

Table S2: Descriptive statistics of targeted individuals regardless of subsequent employment status

Variable	Out of labour market N=123	Remain in labour market N=313	p-value	N
	Mean (SD) for continuous variable otherwise %			
Cognitive function	26.09 (4.29)	27.59 (2.56)	0.001	436
Self-Reported Health:			0.040	436
Bad	16	7.9		
Normal	45	41		
Good	38	44		
Very Good	1.9	6.5		
Best	0	0.3		
Age	64.11 (6.18)	62.47 (5.90)	0.015	436
Female	68	52	0.007	436
Education:			0.005	436
≤ Elementary school	49	29		
Middle school	19	28		
High school	31	36		
≥ College/University	1.4	7.5		
Married:	75	84	0.066	436
Working hours (week)	42.01 (17.29)	46.86 (20.51)	0.055	436
Working days (week)	4.86 (1.28)	4.97 (1.05)	0.4	436
Monthly Income (10,000KRW)	92.32 (41.09)	107.92 (41.99)	0.002	436

Notes: This table describes the means of the observable characteristics comparing individuals with earnings below the minimum wage based on their subsequent employment status. Listed values are mean (standard deviation) for continuous variables and percentages otherwise. Survey sampling weights were used in the summary statistics. P-values indicate statistical differences between the two groups. All values were measured in 2016, prior to the minimum wage hike.

Table S3: Potential mechanisms.

Dependent variable:	Working hours	Income	Job satisfaction	Job security	Drinking	Smoking
MW increase	-4.061^{***}	17.46^{***}	-0.021	0.103	-0.004	0.027
	(1.122)	(4.372)	(0.048)	(0.053)	0.004	0.015
Observations	924	924	922	924	924	924

Notes: This table presents an estimate of the impact of the 2016 to 2018 minimum wage increase on health outcomes. The intervention group comprised participants who reported hourly wages below the minimum wage. Participants reporting hourly wages equal to 100-150% of the minimum wage were selected as the control group. Working hours refer to the weekly working hours. Income is the current monthly salary. Job satisfaction and job security are re-coded into binary variables with 1 indicating positive experience. Drinking and smoking are binary variables representing the current status (Yes/No). All specifications used time-varying variables including pension status, marital status and 5-year age categorisation. The standard errors are reported in parentheses. *$p<0.05$, **: $p<0.01$, ***: $p<0.001$.

Table S4: Descriptive statistics with stringent intervention group

Variable	Intervention group N=60	Control group N=149	p-value	N
	Mean (SD) for continuous variable otherwise %			
Cognitive function	28.00 (2.09)	27.82 (2.65)	0.6	209
Self-Reported Health:			0.13	209
Bad	12	2.4		
Normal	39	33		
Good	39	57		
Very Good	8.1	5.3		
Best	1.3	2.1		
Age	61.47 (5.57)	59.82 (4.31)	0.036	209
Female	57	44	0.14	209
Education:			0.008	209
$\leq$ Elementary school	31	16		
Middle school	16	16		
High School	49	52		
$\geq$ College/University	3.2	16		
Married:	82	84	0.8	209
Working hours (week)	44.81 (11.89)	41.85 (10.07)	0.076	209
Working days (week)	5.23 (0.93)	5.10 (0.88)	0.4	209
Monthly Income (10,000KRW)	116.14 (29.65)	164.07 (42.14)	<0.001	209

Notes: This table describes the means of the characteristics when comparing the intervention and control groups. The intervention group was defined as participants whose new hourly wage after the policy change was between 100-120% of the minimum wage and whose pre-intervention hourly wages were below the minimum wage. Participants reporting hourly wages equal to 100-150% of the minimum wage were selected as the control group. Listed values are mean (standard deviation) for continuous variables and percentages otherwise. Survey sampling weights were used. P-values indicate statistical differences between the two groups. All values were measured in 2016, prior to the intervention.

Table S5: Minimum wage and health - stringent intervention group.

	Stringent sample		Placebo test	
Dependent variable:	Cognitive function	SR health	Cognitive function	SR health
MW increase	-1.02^{*}	0.072	0.340	0.268^{**}
	(0.400)	(0.105)	(0.256)	(0.083)
Observations	418	418	542	542

Notes: This table presents an estimate of the impact of a minimum wage increase on health outcomes. The intervention group was defined as participants whose new hourly wage after the policy change was between 100-120% of the minimum wage and whose pre-intervention hourly wages were below the minimum wage. Participants reporting hourly wages equal to 100-150% of the minimum wage were selected as the control group. Propensity score weights were then applied. Cognitive scores range from 0 to 30, and self-rated health from 1 to 5. Columns 2-3 report large minimum wage increase from 2016 to 2018. Columns 4-5 show the modest increase from 2014 to 2016, which served as the placebo test. All specifications used time-varying variables such as pension status, marital status and 5-year age categorisation. Standard errors are reported in parentheses. *$p<0.05$, **: $p<0.01$, ***: $p<0.001$.

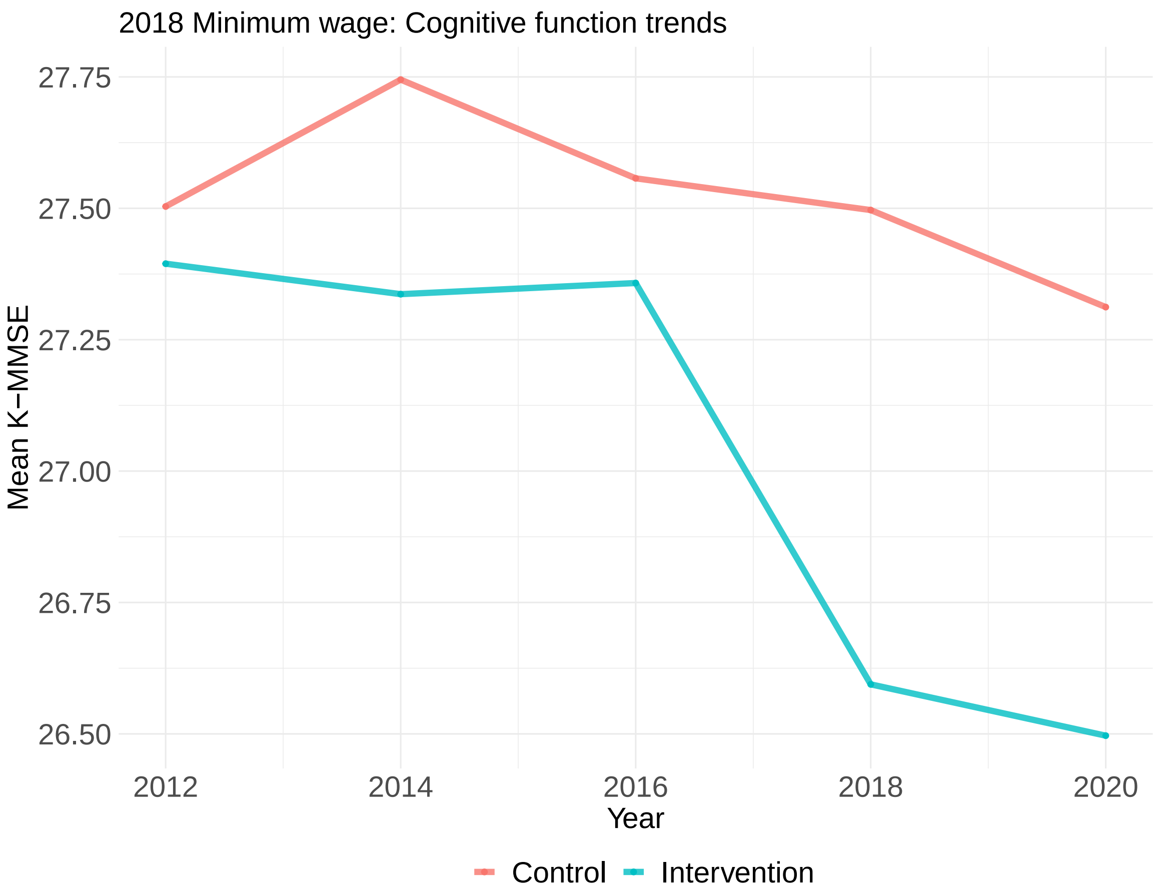

Figure S1: **Trends in mean cognitive functioning** This figure presents the trends in mean cognitive functioning for intervention and control groups for the large minimum wage increase from 2016 to 2018. Cognitive function was measured by the Korean version of Mini-Mental State Examination (K-MMSE). K-MMSE ranges from 0 to 30. The intervention group comprised participants whose reported hourly wages in 2016 were below the new minimum wage. The control group included participants whose hourly wages in 2016 were between 100-150% of the new minimum wage.

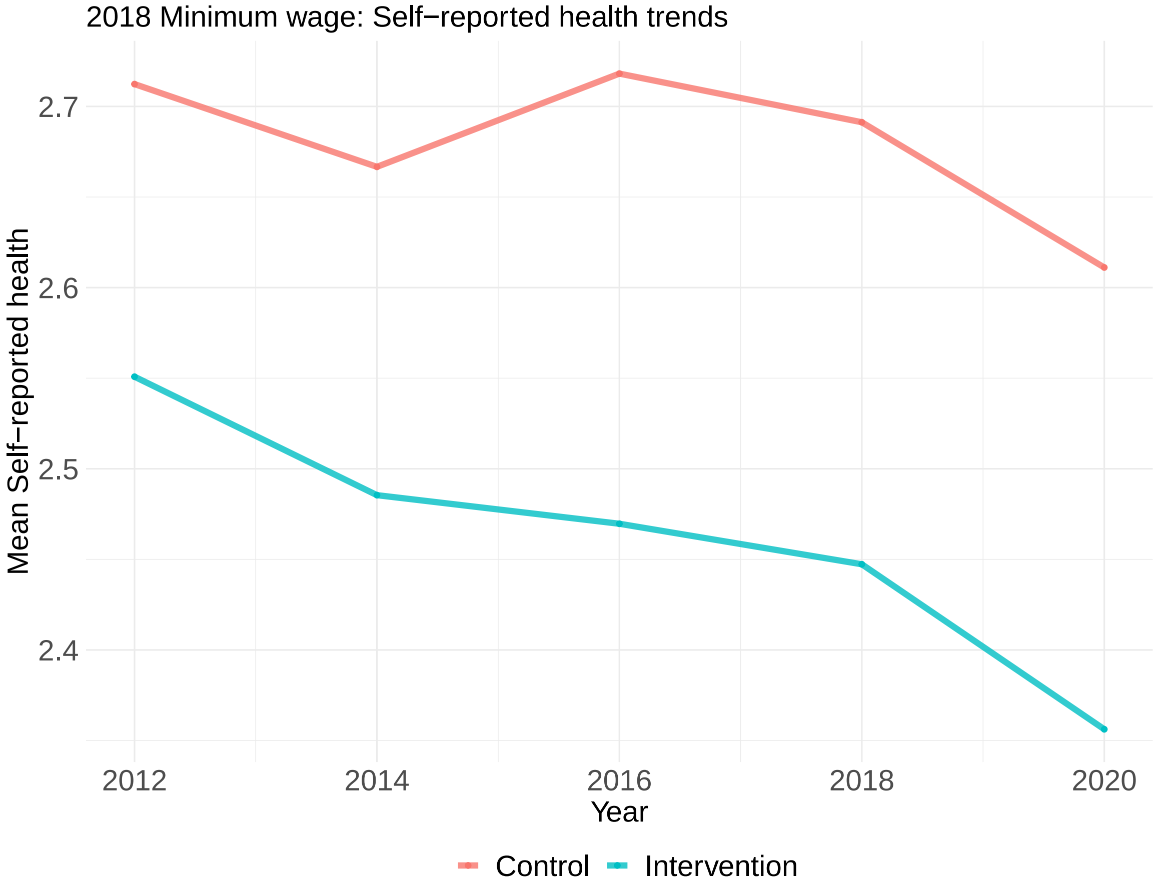

Figure S2: **Trends in mean self-reported health** This figure presents the trends in mean self-reported health for intervention and control groups for the large minimum wage increase from 2016 to 2018. Self-reported health ranges from 0 to 5. The intervention group comprised participants whose reported hourly wages in 2016 were below the new minimum wage. The control group included participants whose hourly wages in 2016 were between 100-150% of the new minimum wage.

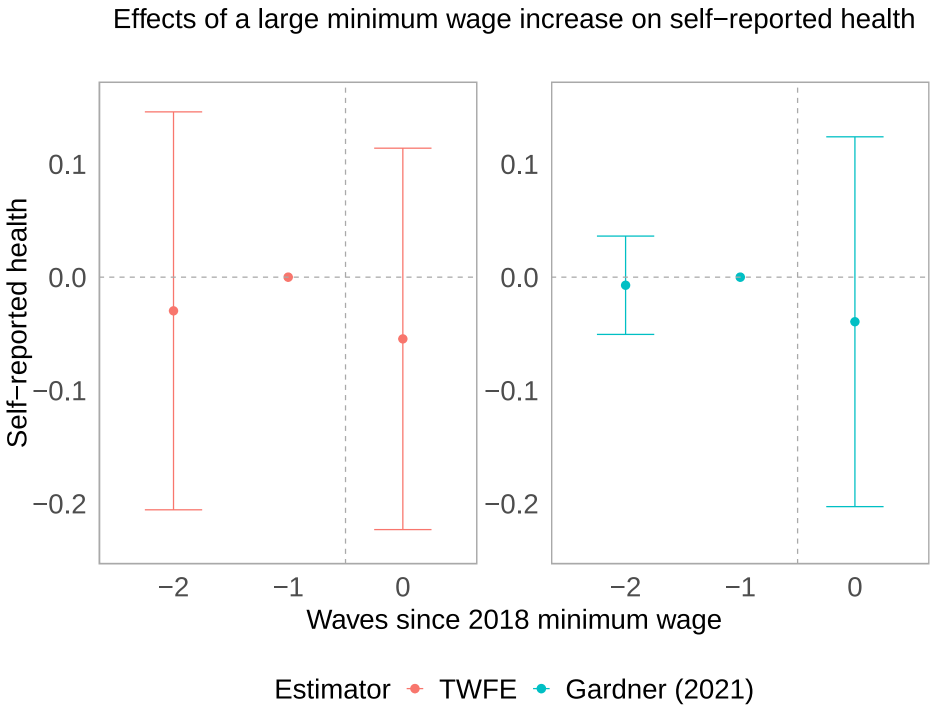

Figure S3: **Event study results regarding self-reported health** These figures present the dynamic effects of the large minimum wage increase from 2016 to 2018 on cognitive functioning. The left panel shows the results from using a two-way fixed effects estimator, while the right panel displays the results from the two-stage difference-in-differences estimator. Self-reported health ranges from 0 to 5. The intervention group comprised participants whose reported hourly wages in 2016 were below the new minimum wage. The control group included participants whose hourly wages in 2016 were between 100-150% of the new minimum wage.
